# The efficacy and safety of chidamide in combination with etoposide and glucocorticoids for the treatment of hemophagocytic lymphohistiocytosis in adult patients: an open-label, single-center study

**DOI:** 10.3389/fimmu.2024.1415597

**Published:** 2024-07-08

**Authors:** Junxia Hu, Jingshi Wang, Zhao Wang

**Affiliations:** Department of Hematology, Beijing Friendship Hospital, Capital Medical University, Beijing, China

**Keywords:** chidamide, etoposide, hemophagocytic lymphohistiocytosis, efficacy, safety

## Abstract

**Background:**

Hemophagocytic lymphohistiocytosis (HLH) is a life-threatening condition characterized by hyperinflammation and organ failure, with a high mortality rate. Current first-line treatments for adult patients have limited efficacy and significant toxicity. The novel selective histone deacetylase inhibitor (HDACi), chidamide, has shown promise in preclinical studies for the potential treatment of HLH.

**Methods:**

An open-label, single-center study was conducted to evaluate the efficacy and safety of chidamide in combination with etoposide and glucocorticoids for the treatment of HLH in adult patients. Seventeen patients who fulfilled at least five of the eight HLH-2004 criteria were enrolled and treated with the combination therapy. The primary outcome was overall response rate (ORR), and secondary outcomes included survival, safety and tolerability, and changes in laboratory indicators.

**Results:**

A total of 17 HLH patients who met the inclusion criteria were enrolled in this study, with a male to female ratio of 1.8:1. The age range at enrollment was 31 to 71 years old, with a median age of 52 years old. The ORR was 76.5% (13/17 patients), with a complete response (CR) rate of 17.6% (3/17 patients) and a partial response (PR) rate of 58.8% (10/17 patients). The median overall survival (OS) was not achieved, with OS at 6 months and 12 months being 81% and 65%, respectively. The median progression free survival (PFS) was not achieved, with PFS at 6 months and 12 months being 68% and 55%, respectively. Hematologic toxicities is the most common. Safety profile was favorable, with very few cases of grade 3/4 toxicities observed. The results showed that the levels of sCD25, platelets, aspartate aminotransferase, lactate dehydrogenase, and albumin in these patients were significantly improved 3 weeks after treatment.

**Conclusion:**

The addition of chidamide to etoposide and glucocorticoids may be a promising new treatment option for patients with HLH, with a high ORR, manageable safety profile, and significant improvement in laboratory indicators. Further research is needed to confirm these findings and determine the optimal dosing and duration of therapy.

## Introduction

Hemophagocytic lymphohistiocytosis (HLH) is a life-threatening condition characterized by excessive immune activation, resulting in inflammatory storms and multi-organ failure (1). HLH can be primary or secondary, with secondary HLH (sHLH) being more common in adult and frequently induced by infectious, autoimmune, and malignant diseases (2). The mortality rate of sHLH is high, ranging from 50-75% (3). Adult patients are treated with regimens that approximate the HLH-94 protocol, but more than 30% of patients do not respond to this treatment (4). While the DEP regimen (combination doxorubicin, etoposide, and methylprednisolone) has shown an overall response rate of 76.2% and a median survival of 28 weeks in refractory adult HLH patients, it cannot achieve long-term stable control of the primary disease (5). Therefore, new treatment options are needed.

Chidamide is a novel selective histone deacetylase inhibitor (HDACi) that has been shown to promote the recovery of normal immune function (6, 7)and treat potential causes (8, 9)of HLH, thereby ending the inflammatory cytokine storm. HDACi can also sensitize etoposide (VP-16) by promoting the activation of caspase 8 and caspase 3, upregulating the expression of topoisomerase II, and promoting the degradation of DNA repair kinases (10). Glucocorticoids can strongly inhibit the activation, differentiation, and chemotaxis of the immune system.

Therefore, we conducted a prospective single-arm clinical study to evaluate the efficacy and safety of chidamide in combination with etoposide and glucocorticoids in HLH patients.

## Methods

We performed an open-label, single-center study to examine the efficacy, safety, and tolerability of chidamide in HLH patients at Beijing Friendship Hospital Affiliated to Capital Medical University. The study was registered at http://www.clinicaltrials.gov (identifier: NCT05137522) and approved by the institutional review board. The protocol followed the principles of the Declaration of Helsinki and the International Conference on Harmonization Guidelines for Good Clinical Practice.

### Patients

We enrolled adult patients (≥18 years) who fulfilled at least five of the eight HLH-2004 criteria for hemophagocytic lymphohistiocytosis (11). Patients with active gastrointestinal bleeding or newly diagnosed thrombotic diseases, a left ventricular ejection fraction of <50%, hepatitis B or C virus infection, or known HIV infection were excluded, as were pregnant or lactating females. Informed consent was obtained from all participants.

### Regimen

Patients meeting the inclusion and exclusion criteria were treated with the following regimen: chidamide (30 mg, po, TIW), etoposide (100 mg/m2, iv, day 1), and methylprednisolone (2 mg/kg, iv, days 1-3; 0.75 mg/kg, iv, days 4-6; 0.25 mg/kg, iv/po, days 7-9; 0.1 mg/kg, iv/po, days 10-21). Cycles of the regimen were repeated every 21 days. Patients with ineffective or serious adverse events were excluded from the group. Effective patients could continue to use this plan for a total of four courses of treatment. Supportive treatments during systemic chemotherapy included prophylactic oral PPI inhibitors, sodium bicarbonate alkalization of urine, calcium carbonate, compound sulfamethoxazole, entecavir, G-CSF, recombinant human interleukin-11 or thrombopoietin, and red blood cell or platelet infusions as needed.

### Assessment of therapy

The primary endpoint was overall response rate (ORR), defined as the proportion of patients who achieved a complete response (CR) or partial response (PR) according to the HLH efficacy evaluation criteria proposed by Qing Zhang et al. (12) and modifications made based on our center’s experience (5). A CR was defined as normalization of all quantifiable symptoms and laboratory markers of HLH, including levels of sCD25, ferritin, and triglyceride; hemoglobin; neutrophil counts; platelet counts; and alanine aminotransferase (ALT). A PR was defined as at least a 25% improvement in 2 or more quantifiable symptoms and laboratory markers by 3 weeks following the regimen. In addition, subjects’ body temperature had to revert to normal ranges in either complete response or partial response. Other observational indicators in the study included liver and spleen size, bilirubin, and albumin. Secondary endpoints included survival, safety and tolerability, and changes in laboratory indicators. Survival time was calculated from the time patients received the therapy until death or the last follow-up date.

### Adverse events and complications

Adverse events and complications were closely monitored during the treatment, including myelosuppression, gastrointestinal reaction, physical state, infection, bleeding, and other adverse drug reactions.

### Survival and follow-up

Survival and follow-up were also assessed. The survival time was calculated from the time patients received this therapy until death or December 31, 2023. As the HLH patients were being treated, their primary underlying diseases were explored by many methods, including genetic testing, pathogen screening, biopsy pathological examination, and immunological inspection. The primary diseases were treated by antitumor chemotherapy, antipathogen treatment, or allogeneic hematopoietic stem cell transplantation, as appropriate.

### Statistical analysis

All statistical analyses were performed using SPSS 22.0 software. Continuous variables were presented as mean ± standard deviation or median and range, depending on the normality of the distribution. Categorical variables were presented as frequencies and percentages. Comparisons between groups were made using independent sample t-tests or Wilcoxon rank-sum tests for continuous variables and chi-square tests or Fisher’s exact tests for categorical variables. Survival was analyzed using the Kaplan-Meier method, and comparisons between groups were made using the log-rank test. A two-sided P-value < 0.05 was considered statistically significant.

## Results

### Patient characteristics

From November 2021 to December 2023, a total of 17 patients with hemophagocytic lymphohistiocytosis (HLH) who met the inclusion and exclusion criteria were enrolled in this study. The cohort consisted of 11 males and 6 females, with a male-to-female ratio of 1.8:1. The age range at enrollment was 31-71 years, with a median age of 52 years. At the time of disease onset, 13 patients presented with pulmonary infections, 5 patients had pleural or peritoneal effusions, and 1 patient had central nervous system involvement.

In terms of etiology, there were 2 cases of EBV-HLH patients. At the time of diagnosis, 15 patients (88%) had not triggers, and one patient with a history of allogeneic hematopoietic stem cell transplantation for acute myeloid leukemia (AML) and one patient with a history of liver malignancy surgery. AML and liver tumor assessment were assessed to be stable. Four patients (24%) were subsequently diagnosed with lymphoma. One patient was subsequently diagnosed with tuberculosis who had a history of throat malignant tumors after radiotherapy and chemotherapy. Three patients (18%) were highly suspected of hematological malignancy, but there was no definitive histopathological evidence. Patient baseline laboratory results are presented in **Table 1**.

**Table 1:** Baseline laboratory findings of 17 HLH patients.

Laboratory tests (units)	Median (interquartile range)
WBC (×10^9^/L)	3.21 (0.74- 14.51)
NEU (×10^9^/L)	1.52 (0.51- 12.76)
HB (g/L)	80 (57- 116)
PLT (×10^9^/L)	86 (5- 358)
TG (mmol/L)	2.05 (1.16- 7.1)
FIB (g/L)	2.23 (0.8- 6.91)
SF (ng/ml)	2998 (152.9- 72668.1)
sCD25 (pg/ml)	14402 (1472-55548)
NK cell activity (%)	13.77 (8-21.18)
ALT (U/L)	67 (5-302)
AST (U/L)	82.9 (12.1-645.8)
LDH (U/L)	776 (235-4060)
TB (μmol/L)	13.6 (5.86-152.7)
DB (μmol/L)	5.57 (2.94-124.8)
ALB (g/L)	29 (21.2-37.9)
Cr (μmol/L)	67 (37-530)
BUN (mmol/L)	5.87 (3.03-30.9)
CRP (mg/L)	34.55 (0.08-127.68)
PCT (ng/ml)	0.47 (0.02-9.84)
ESR (mm/1h)	27 (7-140)
IL-6 (pg/ml)	22.7 (2.1-53.8)
IL-10 (pg/ml)	8.1 (0.7-2309.5)
IL-18 (pg/ml)	1874.3 (55.2-909931.3)
TNF-α (pg/ml)	9.8 (1.9-64.3)
IFN-γ (pg/ml)	21.1 (2.9-943.5)

WBC, White Blood Cell; NEU, Neutrophils; HGB, Hemoglobin; PLT, Platelet; TG, Triglyceride; FIB, Fibrinogen; SF, Serum Ferritin; sCD25, Soluble Interleukin-2 Receptor-Alpha; ALT, Alanine Aminotransferase; AST, Aspartate Aminotransferase; LDH, Lactate Dehydrogenase; TB, Total Bilirubin; DB, Direct Bilirubin; ALB, Albumin; Cr, Creatinine; BUN, Blood Urea Nitrogen; CRP, C-reactive protein; PCT, Procalcitonin; ESR, Erythrocyte sedimentation rate; IL, Interleukin; TNF-α, Tumor necrosis factor-α; IFN-γ, Interferon-γ.

### Efficacy

**Figure 1:**
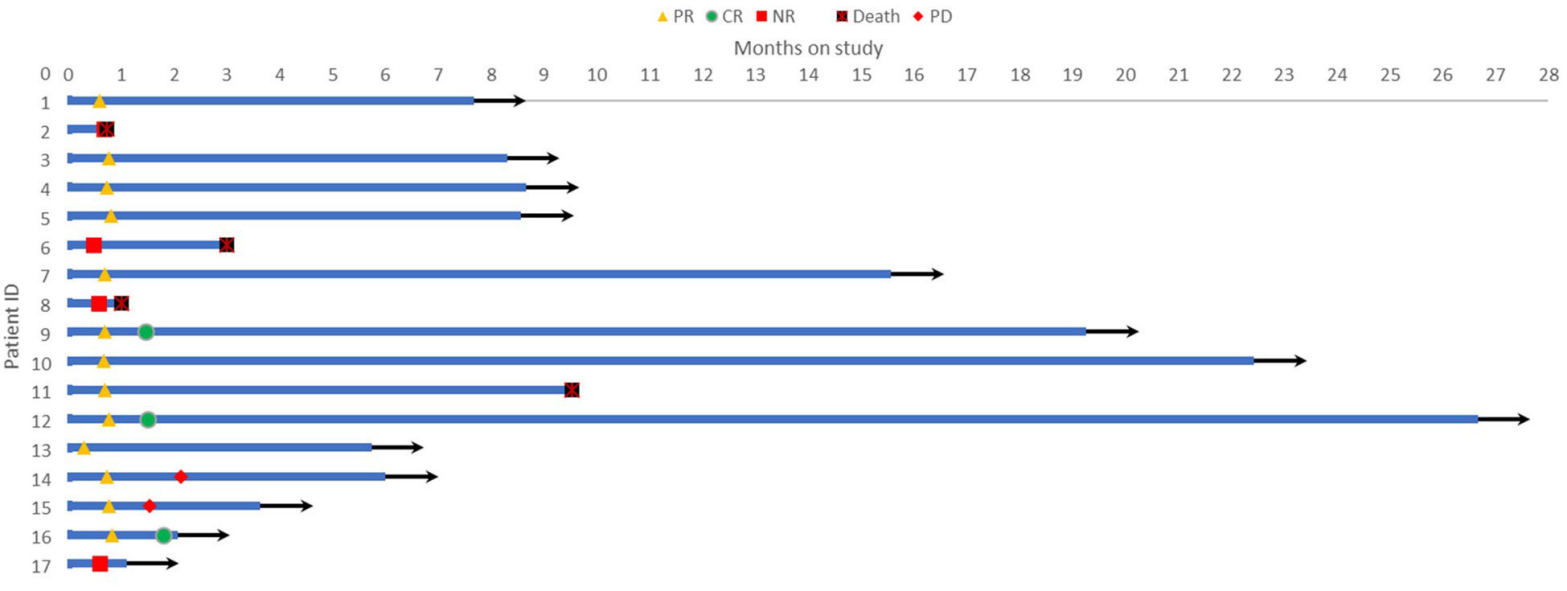
Swimmer plot showing the response status of each patient over time from the start of treatment.

The regimen in this study consisted of a 21-day cycle. All 17 patients received at least one cycle of treatment and were evaluated for efficacy after each cycle of treatment. The results showed that 3 patients (17.6%) achieved complete remission (CR), 10 patients (58.8%) achieved partial remission (PR), of which 2 patients subsequently experienced disease progression, and 4 patients had no response to treatment, resulting in an overall objective response rate (ORR) of 76.5% (**Table 2**). The Swimmer plot in **Figure 1** shows the response status of each patient from the start of treatment over time.

**Table 2:** Efficacy evaluation of enrolled HLH patients.

Response	N(N=17)	Percent(%)	95%CI
CR	3	17.6%	3.8-43.4%
PR	10	58.8%	32.9-81.5%
NR	4	23.5%	6.8-49.9%
PD	2		
ORR	14	76.5%	50.1-93.2%

CR, Complete Response; PR, Partial Response; NR, No Response; PD, Progressive Disease; ORR, Overall Response Rate.

Among the 17 patients, 10 were newly diagnosed, 6 patients were relapsed and one patient was refractory. Of the newly diagnosed patients, 6 achieved PR and 2 achieved CR, resulting in an ORR of 80%. Of the relapsed or refractory patients, 4 achieved PR and 1 achieved CR, resulting in an ORR of 71%.

All patients underwent efficacy evaluation after one cycle of treatment. Thirteen patients achieved PR, 4 were ineffective and withdrew from the study, and 3 patients subsequently found HLH-inducing factors through laboratory tests or pathological results and received corresponding primary disease-related treatment. Ten patients received two cycles of treatment, with 8 achieving PR and 2 achieving CR. Two patients withdrew from the study due to self-perceived weakness and poor compliance, one received lymphoma-related treatment after finding the cause, one was withdrawn due to disease progression, and one achieved CR but developed deep vein thrombosis and pulmonary artery embolism in both lower extremities and underwent surgical treatment. One patient was withdrawn due to disease progression after three cycles of treatment. Four patients completed four cycles of treatment, with 3 achieving PR and 1 achieving CR. One patient was found to have vascular immunoinflammatory T-cell lymphoma during treatment. Five patients had large amounts of pleural/ascites at baseline, which completely disappeared after treatment. Patient demographics and treatment details are presented in **Table 3**.

**Table 3:** Treatment details of 17 HLH patients.

Patient	Gender	Age(year)	Aetiology	Type	cycle of treatment
1	M	43	Unknown, later diagnosed with HSTCL	Refractory	1
2	F	71	Unknown	Newly diagnosed	1
3	F	51	Unknown, history of AML allo-HSCT	Newly diagnosed	2
4	M	43	Unknown	Newly diagnosed	4
5	M	58	Unknown	Newly diagnosed	4
6	F	68	EBV	Newly diagnosed	1
7	M	40	Unknown, later diagnosed as DLBCL	Refractory	1
8	M	48	Unknown, history of liver cancer surgery	Relapsed	1
9	F	50	Unknown	Newly diagnosed	2
10	M	31	Unknown, later diagnosed as indolent PTCL	Relapsed	2
11	M	71	Unknown, later diagnosed as AITL	Relapsed	4
12	F	67	Unknown, DLBCL possible	Newly diagnosed	4
13	M	52	Unknown, later diagnosed as Tuberculosis, history of pharyngeal cancer treatment	Newly diagnosed	1
14	M	67	Unknown, MDS/MPN possible	Newly diagnosed	3
15	M	60	Unknown, B-NHL possible	Newly diagnosed	2
16	F	41	Unknown	Relapsed	2
17	M	56	EBV	Relapsed	1

M, Male; F, Female; HSTCL, Hepatosplenic T-cell Lymphoma;AML, Acute myeloid leukemia; allo-HSCT, Allogeneic Hematopoietic Stem Cell Transplantation; EBV, Epstein-Barr virus; DLBCL, Diffuse Large B-Cell Lymphoma; CA, Cancer; PTCL, Peripheral T-cell Lymphoma; AITL, Angioimmunoblastic T-cell Lymphoma; MDS, Myelodysplastic syndrome; MPN, Myeloproliferative Neoplasms; NHL, Non-Hodgkin Lymphoma.

A comparison of HLH-related laboratory indicators was performed in the 17 HLH patients treated with this regimen before and 3 weeks after treatment, including triglycerides, fibrinogen, serum ferritin, sCD25 level, white blood cell count, neutrophil count, hemoglobin level, platelet count, alanine aminotransferase, aspartate aminotransferase, lactate dehydrogenase, total bilirubin level, and albumin. The results showed that compared with the indicators before treatment, the levels of sCD25, platelets, aspartate aminotransferase, lactate dehydrogenase, and albumin in these patients were significantly improved 3 weeks after treatment, with statistically significant differences (*P* < 0.05) (**Table 4**).

**Table 4:** Changes in clinical indicators before and after treatment in HLH patients.

Indicator	Before treatment	After treatment	*P*-value
TG (mmol/L)	2.05(1.16-7.10)	1.39(0.51-7.75)	0.433
FIB (g/L)	2.23(0.80-6.91)	2.45(0.6-5.26)	0.300
SF (ng/ml)	2998(152.9-72668)	2428.9(94.1-47192.5)	0.245
sCD25 (pg/ml)	14402(1472-55548)	7123(1011-55447)	0.001*
WBC (×10^9^/L)	3.21(0.74-14.51)	3.72(2.47-18.79)	0.975
NEU (×10^9^/L)	1.52(0.51-12.76)	2.73(0.82-17.56)	0.730
HB (g/L)	80(57-116)	79(60-128)	0.706
PLT (×10^9^/L)	86(5-358)	51(4-162)	0.019*
ALT (U/L)	67(5-302)	31(1-142)	0.109
AST (U/L)	82.9(12.1-645.8)	20.9(10.3-155.5)	0.026*
LDH (U/L)	776(235-4060)	211(137-2705)	0.041*
TB (μmol/L)	13.6(5.86-152.7)	20.35(3.73-36.54)	0.778
ALB (g/L)	29(21.2-37.9)	34.2(27.3-39.2)	0.001*

TG, Triglyceride; FIB, Fibrinogen; SF, Serum Ferritin; sCD25, Soluble Interleukin-2 Receptor-Alpha; WBC, White Blood Cell; NEU, Neutrophils; HGB, Hemoglobin; PLT, Platelet; ALT, Alanine Aminotransferase; AST, Aspartate Aminotransferase; LDH, Lactate Dehydrogenase; TB, Total Bilirubin; ALB, Albumin; * *P* < 0.05, statistically significant.

### Survival analysis

**Figure 2:**
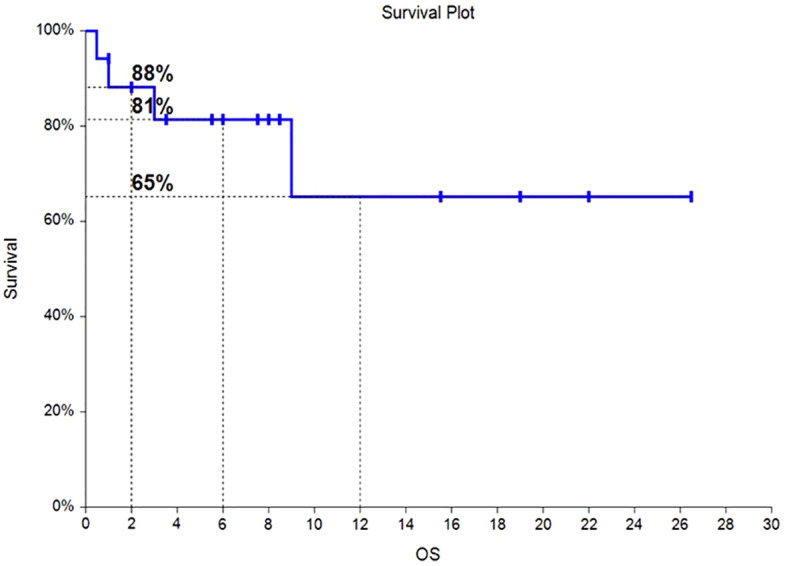
Overall survival curve for HLH patients.

**Figure 3:**
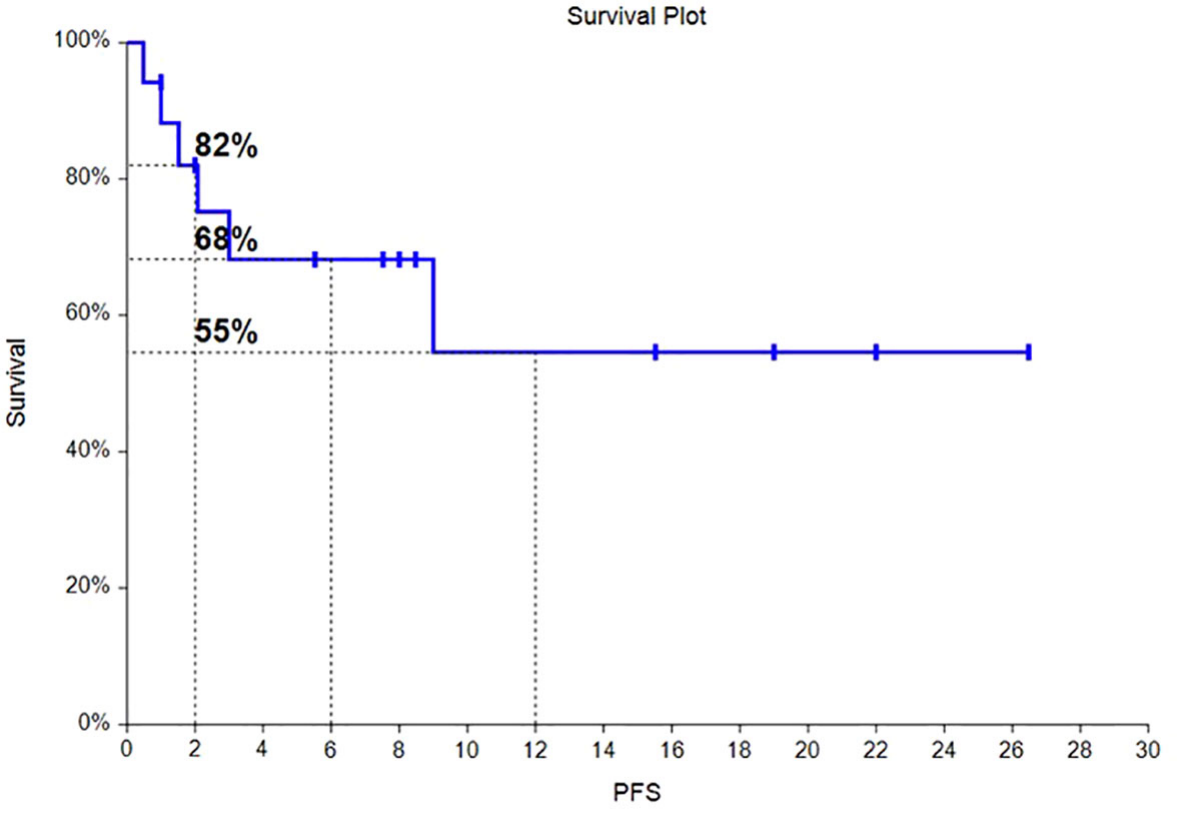
Progression-free survival curve for HLH patients.

Of the 17 HLH patients, from the time of enrollment to death or the last follow-up date of January 31, 2024, 4 patients died, with an overall survival rate of 76.5%. Among them, 2 patients died from progression of the primary disease, 1 patient died from COVID-19 pneumonia infection, and 1 patient died from severe pulmonary infection and multiple organ dysfunction syndrome caused by long-term bedridden status. As of the follow-up date, the median OS had not been reached, and the OS rates at 6 months and 12 months were 81% and 65%, respectively. The survival time curve is shown in **Figure 2**. The median PFS had not been reached, and the PFS rates at 6 months and 12 months were 68% and 55%, respectively. The progression-free survival curve is shown in **Figure 3**.

### Toxicity

Hematology toxicity is the most common during this study. Adverse events (AEs) that occurred in more than 10% of patients were thrombocytopenia (8/17, 47%), leukopenia/neutropenia (6/17, 35%), anemia (2/17, 12%), nausea/vomiting (4/17, 24%) and fatigue (3/17, 18%). Most AEs were grade 1 or 2, and grades 3-4 AEs that occurred in more than 10% of the patients was thrombocytopenia (2/17, 12%). One patient developed severe pulmonary infection which was considered to be related to the patient’s long-term bedridden status and poor general condition, and not clearly related to the drug. One patient had achieved CR, who developed deep vein thrombosis and pulmonary vascular thrombosis during treatment. It could not be determined whether it was related to the drug, and the patient was discontinued from the study and underwent surgical treatment (**Table 5**).

**Table 5:** Adverse events possibly related to chidamide.

Adverse events	All Grade, N (%)	Grade 3-4, N (%)
Leukopenia/Neutropenia	6 (35%)	1 (6%)
Thrombocytopenia	8 (47%)	2 (12%)
Anemia	2 (12%)	1 (6%)
Nausea/Vomiting	4 (24%)	0
AST/ALT Increased	1 (6%)	0
Lung infection	1 (6%)	0
Diarrhea	1 (6%)	0
Bleeding/thrombosis	0	0
Fatigue	3 (18%)	0

ALT, Alanine Aminotransferase; AST, Aspartate Aminotransferase.

## Discussion

Current first-line treatments for adult HLH patients have limited efficacy and significant toxicity. The novel selective histone deacetylase inhibitor (HDACi), chidamide, has shown promise in preclinical studies for the potential treatment of HLH. In this open-label, single-center study, we evaluated the efficacy and safety of chidamide in combination with etoposide and glucocorticoids for the treatment of HLH in adult patients.

Lymphoma-associated hemophagocytic lymphohistiocytosis (LA-HLH) is the most common type (13), but its high malignancy, rapid disease progression, and short survival time make LA-HLH difficult to diagnose. Some patients may not have palpable tumors or enlarged lymph nodes for biopsy, leading to missed diagnoses and classification as unknown HLH. One patient diagnosed with HLH of unknown origin was found to have diffuse large B-cell lymphoma of the spleen after splenic rupture and bleeding following chemotherapy (14). A study of 19 patients with recurrent HLH of unknown origin found that 7 patients were diagnosed with lymphoma after splenectomy (15). In this study, 15 of 17 patients (88%) were diagnosed with unknown HLH, 4 patients (24%) were subsequently diagnosed with lymphoma during follow-up or treatment, one patient was subsequently diagnosed with tuberculosis who had a history of throat malignant tumors after radiotherapy and chemotherapy, while 3 patients (18%) were highly suspected of hematological malignancies but lacked definitive histopathological evidence. Therefore, patients with HLH of unknown origin should undergo regular follow-up and repeat biopsies as necessary to assist in diagnosis and guide subsequent treatment.

Based on the HLH-94 regimen, the 5-year survival rate has improved from 5% to over 50% (16), and may require alternative treatment options, including other immunosuppressive chemotherapy and/or biologic agents (17). Although this study achieved an ORR of 76.2% for R/R-HLH, the overall mortality rate was 54% due to progression of the underlying disease or recurrence of HLH (5). In our study, the overall response rate (ORR) was 76.5% (13/17 patients), with a complete response (CR) rate of 17.6% (3/17 patients) and a partial response (PR) rate of 58.8% (10/17 patients). The results showed that the levels of sCD25, platelets, aspartate aminotransferase, lactate dehydrogenase, and albumin in these patients were significantly improved 3 weeks after treatment (P < 0.05). A study suggests that (18), HDACi reduces the excessive infiltration of neutrophils and macrophages in tissues by regulating the acetylation levels of NF-κB in the heart and liver. The levels of ALT, AST, LDH, and inflammatory factors were significantly reduced, reducing the severity of liver and heart damage. Meanwhile, flow cytometry analysis showed a significant decrease in the percentage of T lymphocytes (CD3+) in the spleen.

Of the 17 HLH patients, from the time of enrollment to death or the last follow-up date of January 31, 2024, 4 patients died, with an overall survival rate of 76.5%. The median overall survival was not achieved, with OS at 6 months and 12 months being 81% and 65%, respectively. The median progression free survival (PFS) was not achieved, with PFS at 6 months and 12 months being 68% and 55%, respectively. Hematologic toxicities is the most common. Safety profile was favorable, with very few cases of grade 3/4 toxicities observed. Chidamide has also achieved similar results in multiple clinical trials of lymphoma (19–21). Perhaps chidamide can be a new treatment option for HLH patients.

**Figure 4:**
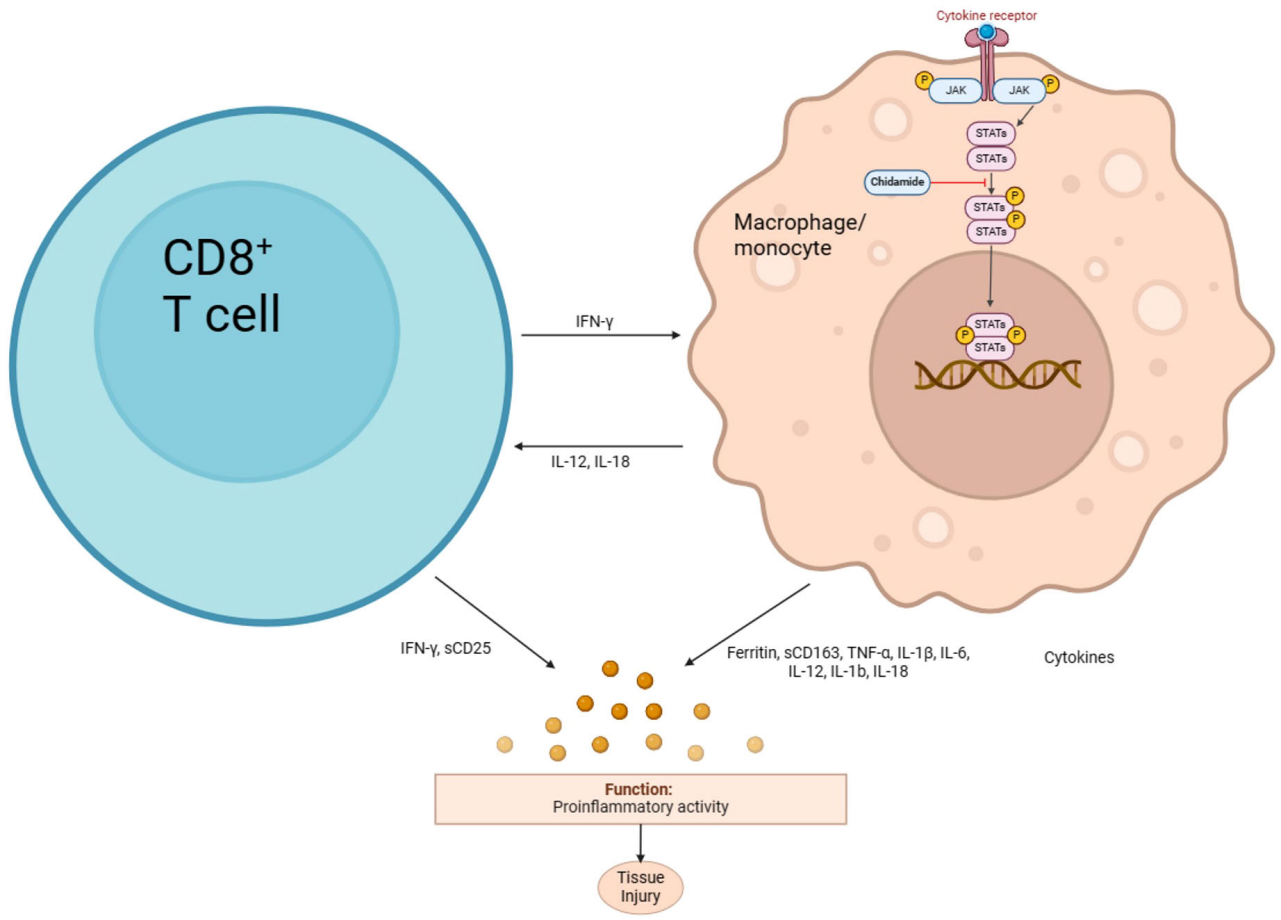
Hypothesis diagram of the mechanism of chidamide in HLH.

Chidamide is an oral selective histone deacetylase (HDAC) inhibitor that selectively inhibits HDAC1, HDAC2, HDAC3, and HDAC10, inducing apoptosis and growth arrest in leukemia cells. It has been approved for the treatment of relapsed or refractory peripheral T-cell lymphoma (R/R PTCL) (22) and is currently being studied for use in other leukemia and myelodysplastic syndromes (MDS) (23, 24). Chidamide’s anti-inflammatory effect may be due to its ability to inhibit the NF-κB signaling pathway, which is involved in the body’s inflammatory and immune responses (**Figure 4**). HDAC3, in particular, plays a crucial role in the deacetylation of p65 (25). In traumatic spinal cord injury-induced inflammation (26), Valproic acid (VPA) treatment attenuated the inflammatory response by modulating microglia polarization through STAT1-mediated acetylation of the NF-κB pathway, dependent of HDAC3 activity. The HDAC3 inhibitors protected wild-type or Hipk2 BMs-reconstituted mice from LPS-induced endotoxemia (27). Mechanistically, HIPK2 phosphorylated HDAC3 to inhibit its enzymatic activity, thus reducing the deacetylation of p65 to suppress NF-κB activation, restraining excessive inflammation. Another study revealed that HDAC3-deficient macrophages fail to activate nearly half of LPS-induced inflammatory gene expression (28), highlighting the co-activating role of HDAC3 in NF-κB-dependent gene expression.

The combination of chidamide with etoposide, a cell cycle-specific chemotherapeutic agent, is being investigated in clinical trials for the treatment of hematological malignancies, which has shown high tolerability and improved chemotherapy efficacy (29). The anti-inflammatory effect of chidamide and its potential to sensitize etoposide make this combination a promising approach for the treatment of inflammatory diseases and cancers. Overall, chidamide’s ability to inhibit HDAC3 and subsequently suppress NF-κB signaling and inflammatory gene expression highlights its potential as a therapeutic agent for inflammatory diseases and cancers.

In conclusion, the addition of chidamide to etoposide and glucocorticoids may be a promising new treatment option for patients with HLH, with a high ORR, manageable safety profile, and significant improvement in laboratory indicators. Further research is needed to confirm these findings and determine the optimal dosing and duration of therapy. The limitations of this study include the small sample size and the lack of a control group. Despite these limitations, this study provides valuable insights into the potential role of chidamide in the treatment of HLH.

## Data availability statement

The original contributions presented in the study are included in the article/supplementary material. Further inquiries can be directed to the corresponding author.

## Ethics statement

The studies involving humans were approved by the Ethics Committee of Beijing Friendship Hospital. The studies were conducted in accordance with the local legislation and institutional requirements. The participants provided their written informed consent to participate in this study. Written informed consent was obtained from the individual(s) for the publication of any potentially identifiable images or data included in this article.

## Author contributions

JH: Writing – original draft, Writing – review & editing. JW: Writing – review & editing. ZW: Writing – review & editing.
